# A decrease in aquaporin 2 excretion is associated with bed rest induced high calciuria

**DOI:** 10.1186/1479-5876-12-133

**Published:** 2014-05-19

**Authors:** Grazia Tamma, Annarita Di Mise, Marianna Ranieri, Maria Svelto, Rado Pisot, Giancarlo Bilancio, Pierpaolo Cavallo, Natale G De Santo, Massimo Cirillo, Giovanna Valenti

**Affiliations:** 1 Department of Biosciences, Biotechnologies and Biopharmaceutics, University of Bari Aldo Moro, Via Amendola 165/A, Bari 70126, Italy; 2 University of Primorska, Primorska, Slovenia; 3 University of Salerno, Salerno, Italy; 4 Department of Medicine, Second University of Naples, Naples, Italy

**Keywords:** Aquaporin-2, Hypercalciuria, Bed rest, Microgravity

## Abstract

**Background:**

Exposure to microgravity or immobilization results in alterations of renal function, fluid redistribution and bone loss, which couples to a rise of urinary calcium excretion. We recently demonstrated that high calcium delivery to the collecting duct reduces local Aquaporin-2 (AQP2) mediated water reabsorption under vasopressin action, thus limiting the maximal urinary concentration and reducing calcium saturation. To investigate renal water balance adaptation during bed rest, a model to mimic the effects of microgravity on earth, the effect of changes in urinary calcium on urinary AQP2 excretion were assessed.

**Methods:**

Ten healthy men (aged 21-28 years) participated in the experiment. Study design included 7 days of adaptation and 35 days of continuous bed rest (days -6 to 0 and 1 to 35, respectively) under controlled diet. Food records and 24-hour urine samples were collected daily from day -3 to 35. Changes in blood hematocrit were used as an indirect index of plasma volume changes. AQP2 excretion was measured by ELISA.

**Results:**

Bed rest induced bone demineralization and a transient increase in urinary calcium followed by transient decrease in AQP2 excretion, which can reduce the urine concentrating ability causing plasma volume reduction. The return of calciuria to baseline was followed by a recovery of AQP2 excretion, which allows for a partial restoration of plasma volume.

**Conclusions:**

These results further support the view that urinary calcium can modulate the vasopressin-dependent urine concentration through a down-regulation of AQP2 expression/trafficking. This mechanism could have a key role in the prevention of urine super-saturation due to hypercalciuria.

## Background

Urinary calcium saturation is an important factor in nephrolitiasis and is strictly correlated to vasopressin-dependent water reabsorption in the kidney. Vasopressin signaling promotes water reabsorption in the renal collecting duct by triggering redistribution of the water channels AQP2 from intracellular vesicles into the plasma membrane [1-3]. This permits water entry into the cell and water exit through basolateral AQP3 and AQP4 resulting in the concentration of urine. Defects of AQP2 trafficking cause diseases such as nephrogenic diabetes insipidus (NDI), a disorder characterized by a massive loss of hypoosmotic urine [4]. AQP2 is partially excreted in the urine [5] and excretion is proportional to its expression in the kidney and in the luminal membrane of renal collecting duct principal cells making a useful biomarker for water concentration diseases [6,7]. Specifically, an increase in AQP2 excretion is observed under vasopressin action as a result of AQP2 activation and translocation to the membrane whereas a reduced AQP2 excretion reflects reduced activation of the water channel and reduced renal ability to conserve water. We have previously shown that in hypercalciuric subjects the physiological response to vasopressin is followed by a less pronounced increase in urinary osmolality and urinary AQP2 indicating a reduced urinary concentrating ability [8]. These data suggest that high calcium delivery to the collecting duct reduces local AQP2 mediated water reabsorption under vasopressin action limiting the maximal urinary concentration and reducing calcium saturation.

Immobilization or exposure to microgravity induce several alterations of renal function including fluid redistribution and bone loss causing increased calciuria and reduced plasma volume [9,10]. In this context, bed rest represents a valuable experimental model to mimic the effects of microgravity on earth [11].

In this work we evaluated the effects of the bed rest-induced changes in urinary calcium on AQP2 excretion. The prior hypothesis was that bed rest-induced increase in urinary calcium should be followed by a decrease in urinary AQP2.

## Methods

### Subjects and bed rest design

In 2007 a 35-day bed rest experiment was performed in the Valdoltra Orthopedic Hospital (Ankaran, Slovenia) as part of the OSMA project (OSteoporosis and Muscular Atrophy), sponsored by the Italian Space Agency (Agenzia Spaziale Italiana, ASI). The study conformed to the Declaration of Helsinki, was approved by the Slovenian National Committee for Medical Ethics at the Ministry of Health (Republic of Slovenia), and included a written informed consent.

Ten healthy men (ages 21-28 years) were selected for the study after medical examinations and routine check-up for exclusion of chronic diseases.

Participants were admitted in the hospital 1 week before for pre-bedrest tests and adaptation. During this period, participants had to sleep in the hospital, had to eat only the food of the hospital diet, had to undergo the pre-bedrest tests and, for the remaining time, were allowed to continue their habitual activities without restraints. Conversely, physical activity was not permitted at any time during bed rest (from day 1 to day 35).

Adherence to the protocol was ascertained by continuous video surveillance and medical supervision. Diet was calculated by the FAO/WHO equations on the basis of the expenditure at rest [12]. Diet was activity-adjusted setting the total calorie intake at 1.4-time the rest expenditure during adaptation and 1.2-time the rest expenditure during bed rest, respectively. The percentages of carbohydrates, fats, and proteins were identical in the two dietary regimens (60%, 25%, and 15%, respectively). The bed rest diet contained approximately 1 g of calcium, 1.3 g of phosphorus, and 600 IU of vitamin D per day.

24-hours urine was accurately collected in the last 4 days of the adaptation week (from day -3 to day 0) and in all the 35 days of bed rest. Morning fasting blood samples and anthropometric data were collected before bed rest at day 0 and weekly during bed rest at days 7, 14, 21, 28 and 35. Anthropometric data included the assessment of body weight, fat mass and fat-free mass by bio-electric impedance (Akern, Florence, Italy). Bone loss was assessed by peripheral quantitative computer tomography (pQCT, XCT 2000, Stratec Medizintechnik, Pforzheim, Germany) 2 days before bed rest (day -2) and on the last day of bed rest (day 35) as described previously [13].

Automated biochemistry and commercially available kits were used for measurements of several variables with inclusion of serum total calcium, urinary calcium, and blood hematocrit used as index of changes in plasma volume.

### Urinary AQP2 measurements by ELISA (enzyme–linked –immunoSorbent –assay)

Urinary AQP2 excretion was measured in the 24-hour urine samples by ELISA as previously described [8]. Approximately 3% of the vasopressin regulated water channel AQP2 expressed in renal collecting duct principal cells is excreted into urine through exosomes, small vesicles (40–80 nm) secreted into the urine by renal epithelial cells. Exosomes remain in the supernatant at standard centrifugation speeds providing an explanation for the finding that AQP2, an integral membrane protein, is plentiful in urines and its excretion increases in response to vasopressin. Briefly, urine samples were spun at 3,000 rpm for 10 min at 4°C to remove cellular debris in the presence of the following protease inhibitors: 2 mM phenylmethylsulfonyl fluoride, 1 mg/ml leupeptin, 1 mg/ml pepstatin. 5 μl of urine sample were diluted to 50 μl in PBS containing 0.01% SDS, placed in a MaxiSorp 96-well microplate (http://www.nuncbrand.com) and incubated for 16 hours at 4°C. In parallel wells, increasing concentrations (50, 100, 200, 300, 400, 500 and 1,000 pg/50 μl) of a synthetic peptide reproducing the last 15 amino acids of the C-terminal region of human AQP2 were incubated as internal standard. Each sample was analyzed in triplicate. Wells were washed with washing buffer (PBS-0.1% Tween20) and incubated with a blocking solution of PBS containing 3% BSA at room temperature for 1 hour. 10 μg of affinity-purified anti-AQP2 antibodies were diluted in blocking solution (final antibody dilution 1:1,000) and 50 μl of the solution was added to each well and incubated for 3 hours at 37°C. Wells were then washed with washing buffer and anti-rabbit IgG conjugated with horseradish peroxidase (http://www.sigmaaldrich.com) was added to each well and incubated for 1 hour at 37°C. After five washings with washing buffer, 50 μl of the substrate solution [2,29-azino-bis(3-ethylbenzthiazoline-6-sulfonic acid); http://www.sigmaaldrich.com] were added to each well and incubated for 30 minutes in the dark. Absorbance was measured with a microplate reader (Model 550, http://www.bio-rad.com) at 405 nm. Urinary AQP2 excretion was expressed as fmol/mg urine creatinine.

### Statistics

The analysis was based on intra-individual comparisons between data at given days of bed rest and pre-bed rest data, from here-on defined as *baseline*. For urinary calcium, baseline was defined as the mean of 24-hours urinary excretion in the last four days of adaptation (days -3, -2, -1, and 0 respectively). For plasma variables, baseline was defined as the value at day 0. For AQP2 excretion baseline was the value measured at day 0. The significance of the bed rest effects was assessed by comparison of bed rest data to baseline by paired *t*-test.

## Results

In this study we evaluated the effect of the increase in urinary calcium associated with prolonged bed rest, on excretion of the AQP2 water channel, a biomarker for collecting duct responsiveness to vasopressin.

Ten healthy men (aged 21-28 years) participated in the experiment in the Hospital of Ankaran (Slovenia). Food records and 24-hours urine samples were collected daily from day -3 to 35. Under these conditions, previous studies showed that bed rest induces bone demineralization and transient increase in urinary calcium [13,14]. Changes in blood hematocrit were used as indirect index of plasma volume changes.

**Figure 1:**
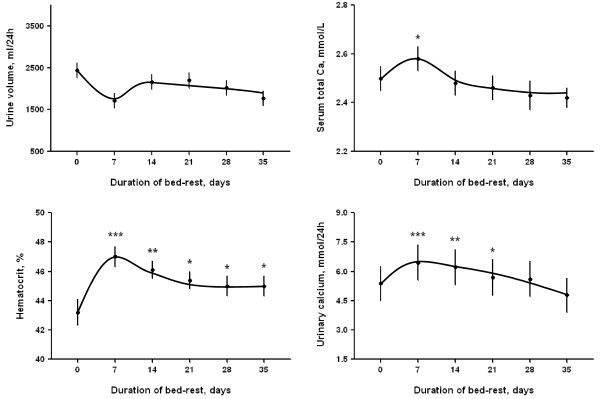
**Time course of urine volume (mL/24 h), serum total calcium (mmol/L), hematocrit (%), urinary calcium (mmol/24 h) during 35 days continuous bed rest.** Data are expressed as mean ± SE. Statistical analysis was done using paired *t*-test (***P < 0.001, **P < 0.01, *P < 0.05).

Urine volume decreased after the switch from adaptation diet to bed rest diet but did not significantly change during bed rest (upper left panel of Figure 1). Bed rest was associated with an increase in blood hematocrit with a transient peak at day 7 and a later stable plateau (lower left panel of Figure 1) indicating a plasma volume contraction. Bed rest associated initially with parallel increases in serum and urinary calcium and later with a slow decline below baseline for serum calcium and toward baseline for urinary calcium (right panels of Figure 1).

Parathyroid hormone (PTH) progressively declined during bedrest reaching a significantly lower value at day 35 with respect to day 0 (29.6 ± 3.6 pg/mL at day 0 *vs* 14.8 ± 2.1 pg/mL at day 35, P < 0.001). The sustained downregulation of the PTH during immobilization was not due to high calcemia which disappeared after day 14 (Figure 1).

**Figure 2:**
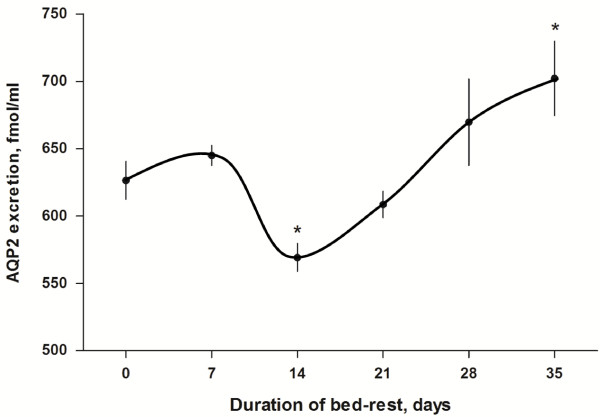
**Mean ± SE of AQP2 excretion during 35 days bed rest.** AQP2 excretion, expressed in fmol/ml, was measured by ELISA in 24 hours urine samples. Statistical analysis was done using paired *t*-test (*P < 0.05).

Evaluation of urinary AQP2 excretion is shown in Figure 2. No significant changes in AQP2 excretion during the first 7 days of bed rest were observed (626 ± 14 fmol/ml and 645 ± 7 fmol/ml at day 0 and day 7 respectively). After the seventh day, however, a significant progressive decrease in AQP2 excretion was measured reaching the lowest value at day 14 (569 ± 10 fmol/ml, P < 0.05 *vs* day 0) followed by a progressive slow recovery. AQP2 remained, however, below baseline values until days 20-22 (608 ± 10 fmol/ml at day 21) (Figure 2). Interestingly, the observed reduction in AQP2 excretion coincided with the transient increase in calciuria in the same given time period, followed by stabilization to baseline values around day 28 (Figures 1 and 2). AQP2 excretion at day 35 that is after return of calciuria to baseline was significantly higher than baseline (702 ± 27 fmol/ml, P < 0.05 *vs* day 0). This increase could reflect late adaptation secondary to plasma volume reduction.

## Discussion

Bed rest represents a good model of simulated microgravity on earth. Previous studies showed that bed rest induces several alterations of physiological parameters including the increase in blood hematocrit due to hemoconcentration and high urinary calcium secondary to bone demineralization [13,14]. The increase in blood hematocrit due to hemoconcentration may reflect water loss associated with a reduction of AQP2-mediated water reabsorption as a consequence of the increase in urinary calcium. Previous evidence supports the notion that this physiological adaptation is due to a complex interplay between AQP2 and the Calcium Sensing Receptor (CaSR) expressed in the luminal membrane of renal collecting duct principal cells [6,8,15-17]. Specifically, during vasopressin action, an increase in urinary calcium concentration would activate the CaSR, which in turn attenuates AQP2-mediated water reabsorption and urinary concentration in humans [8]. This complex mechanism may represent an internal renal defense to mitigate the effects of hypercalciuria on the risk of calcium precipitation during antidiuresis.

The present study would favor this concept demonstrating that bed rest induces a biphasic response in AQP2 excretion. The early phase of bed rest from day 7 to day 14 was associated with a transient significant decrease in AQP2 excretion, which is likely secondary to the hypercalciuric response. This down-regulation of AQP2 excretion could reduce the urine concentrating ability and, via this effect, could contribute to hemoconcentration due to a reduction of plasma volume and consequent increase in hematocrit. In a later phase, the decline of calciuria toward baseline is followed by a recovery of AQP2 excretion, which increases to values significantly higher than baseline at day 35. This late increase in AQP2 could reflect the combination of an increased vasopressin secretion due to plasma volume reduction with near-normal levels of urinary calcium.

Together these data represent the first detailed analysis of the inverse modulation of AQP2 excretion correlated with the transient increase in calciuria during a prolonged bed rest, mimicking *chronic* adaptation to microgravity. A previous study evaluated AQP2 excretion during thermoneutral water immersion (6 hours water immersion), another ground-based analog of microgravity that mimicks, however, *acute* adaptation to microgravity. Water immersion resulted in a significant increase in urinary output that apparently was not related to urinary AQP2 alteration and mainly due to reduced vasopressin secretion [18].

Altogether, our data demonstrate that chronic adaptation to microgravity simulated by prolonged bed rest induces a transient increase in urinary calcium and modulates AQP2 excretion due to the inhibition of AQP2 insertion into the plasma membrane during vasopressin action. A complete recovery of AQP2 excretion is, on the other hand, associated to return of calciuria to normal values.

## Conclusions

In conclusion, we report here that prolonged bed rest induces bone demineralization and transient increase in urinary calcium followed by a transient decrease in AQP2 excretion, which can reduce the ability to concentrate urine ability causing plasma volume reduction. The return of calciuria to baseline is followed by a recovery of AQP2 excretion, which allows for a partial restoration of plasma volume. These results further support the view that urinary calcium can modulate the vasopressin-dependent urine concentration through a down-regulation of AQP2 expression/trafficking. This mechanism could have a key role in the prevention of urine super-saturation due to hypercalciuria.

## Abbreviations

AQP2: Aquaporin 2; CaSR: Calcium sensing receptor; ELISA: Enzyme-linked-immunoSorbent-assay, PTH, Parathyroid hormone.

## Competing interests

The authors declare that they have no competing interests.

## Authors’ contributions

GT, ADM and MR performed the AQP2 measurements and contributed to the analysis of the data. RP organized the bed rest. GB and PC measured the clinical parameters and performed the data analysis. The first drafts of the paper were written by GV and MC. NGDS and MS contributed to the final version of the article. All authors read and approved the final manuscript.
